# The relocation of a psychiatric crisis intervention unit from a somatic to a psychiatric hospital: a multi-method observational study

**DOI:** 10.1038/s41598-026-57920-5

**Published:** 2026-06-19

**Authors:** Janina Billian, Julian Möller, Lukas Imfeld, Franziska Rabenschlag, Philipp Sterzer, Undine E. Lang, Roselind Lieb, Christian G. Huber

**Affiliations:** 1 Klinik für Erwachsene, Universitäre Psychiatrische Kliniken Basel, Basel, Switzerland; 2 Faculty of Psychology, Division of Clinical Psychology and Epidemiology, University of Basel, Basel, Switzerland; 3 Institute for Evaluation Research, Basel, Switzerland

**Keywords:** Crisis intervention, Mental health crisis, Mental health service, Psychiatry stigma, Healthcare transition, Setting effect, Referral, Comparison, Mixed-method, Diseases, Health care, Medical research, Psychology, Psychology

## Abstract

The Crisis Intervention Ward relocated from a general hospital to a psychiatric hospital in Basel, Switzerland, on May 26, 2023. We hypothesize that this relocation influenced patient demographics, frequencies of main diagnoses, involuntary admissions, transfer rates, clinical outcomes, patient satisfaction, and clinician perceptions of the treatment setting. We compared two patient cohorts: one from a 12 month period pre-relocation and the other from a 12 month period post-relocation. Two outpatient clinician surveys—one conducted pre- and one post-relocation—assessed location ratings, treatment satisfaction, and the perceived benefits and disadvantages of each location. Prior to relocation, 625 cases were treated; after relocation, 686 cases. Patient’s age and gender showed no differences. The frequency of affective disorders (F3) was lower (Φ = − 0.124) at the psychiatric hospital. Clinical outcomes, involuntary patient admissions, and transfer rates to other psychiatric wards were comparable. Patient-reported satisfaction was lower at the psychiatric hospital (mean 4.6 vs. mean 5.2, *r* = 0.23). Outpatient clinicians favored the general hospital setting strongly (*r* = 0.78–0.81). Clinical outcomes remained stable, but patient satisfaction was lower after relocation, and clinicians perceived barriers related to accessibility and stigma at the psychiatric hospital.

**Supplementary Information:**

The online version contains supplementary material available at 10.1038/s41598-026-57920-5.

## Introduction

Crises are a universal aspect of human experience, potentially affecting individuals across all levels of society and not limited solely to psychiatric populations. In the field of psychiatry, the concept of “crisis” has traditionally been defined as an acute psychological episode in individuals with previously stable mental health, often triggered by significant psychosocial stressors such as grief, separation, illness, or job loss that exceed their coping capacities^1–4^. In contemporary practice, the term is frequently extended to describe situations where individuals with pre-existing mental disorders are on the verge of decompensation^3,5,6^.

Typically, crises are transient, lasting from several hours to a few weeks^1,7^. During such episodes, individuals may experience substantial disruptions in emotional stability and self-esteem, often accompanied by feelings of anxiety, helplessness, despair, and a perceived loss of control^8,9,4^. People respond to crises through various coping mechanisms, which can be either adaptive, thereby facilitating recovery, or maladaptive, leading to symptom exacerbation or deterioration^8,7^. The ability to navigate daily challenges may become impaired, and other life domains can suffer, especially when maladaptive strategies such as substance abuse or self-harm are employed^10–12^. If unresolved, a crisis can escalate, increasing the risk of suicidal behavior or aggressive outbursts^13,8,14,4,15,16^.

Professional crisis intervention plays a crucial role in preventing such adverse outcomes. It aims to strengthen individuals’ coping strategies and, when necessary, their support networks, and promoting mental health stabilization with the overarching goals of suicide and violence prevention^17,8,9,4^. In many cases, it may be beneficial to provide individuals in crisis with a protected environment within an inpatient setting, which often serves as the first access point to psychiatric and psychotherapeutic care^18,3,19^. Crisis intervention facilities are characterized by their low-threshold access and rapid response capabilities, as timely intervention can mitigate the progression of symptoms, reduce self-harm risks, and foster recovery in a supportive setting^20,7,4^. Inpatient crisis intervention typically lasts five to seven days and involves medical consultations, individualized care planning, counseling, and a variety of therapeutic interventions^18,16^.

This paper focuses on the *Crisis Intervention Ward* (Kriseninterventionsstation; KIS) at the University Psychiatric Clinics (UPK) Basel^21,16^. The KIS offers immediate, low-threshold support for adults in the Basel-City area facing acute mental health crises with various psychiatric symptoms, including suicidal ideation, provided by a multidisciplinary team consisting of doctors, nurses, and social workers^21,16^. Patients can access the KIS directly through a triage process or via referral from general practitioners, psychiatrists, psychotherapists, emergency departments, or community mental health services. In May 2023, following the termination of a rental agreement with the University Hospital Basel (USB), a general hospital located in the city center, the KIS relocated to the campus of a psychiatric institution, the UPK, on the outskirts of Basel. This relocation has prompted questions and criticism regarding the effects of ward location on patient demographics, referral patterns, and treatment outcomes, as well as the broader impact on community perceptions of psychiatric services, such as mental health stigma^22,23^; Regierungsrat des Kantons Basel-Stadt^24–27,16^.

Historically, the placement of psychiatric services has been a subject of ongoing debate^28,29^. In the 19th century, the dominant approach was to establish mental health wards in rural areas, under the belief that tranquility would facilitate recovery^30–32^. Conversely, urban integration initiatives, such as Griesinger’s “Stadtasyle,” aimed to improve access and reduce social isolation^33–35^. Throughout the 20th century, the deinstitutionalization movement shifted the focus toward community-based care, driven by advances in psychopharmacology and evolving societal attitudes^28,36,29^. The goal was to reduce long-term hospitalization and combat stigma^37,29^. Despite this shift, discussions about the optimal location for psychiatric services continued, raising questions about whether proximity to general medical wards enhances accessibility and normalizes treatment, or whether separate, specialized facilities like dedicated psychiatric campuses serve therapeutic needs more effectively^38,39,20,40–45^.

Modern considerations involve balancing accessibility with societal acceptance. Evidence regarding how location influences patient demographics, engagement, outcomes, and public attitudes remains limited and presents mixed results, yet understanding these factors is vital for optimal service design^22,23,46,47,40,48–50^. For example, integrating psychiatric units within general hospitals has been linked to reduced stigma, though findings are not always consistent^25,27^. Similarly, setting can influence patient profiles, including gender, age, diagnosis, and involuntary admission rates, as well as societal attitudes toward mental health^47,51–53,37^.

The recent relocation of the KIS in Basel exemplifies these ongoing debates. Critics argue that moving to the psychiatric campus on the outskirts of the city may hinder access for vulnerable populations and increase societal stigma, while supporters believe that specialized environments could foster better therapeutic outcomes. Public opposition from organizations such as the Basel Psychiatry Commission underscores societal concerns about accessibility and stigma (Basel-City 2023)^26^. This publicly expressed negative stance and emphasis on the disadvantages of the relocation may have influenced public perception and potential patients’ attitudes toward the KIS and the psychiatric hospital overall^54,55^.

Ultimately, the debate over psychiatric ward placement involves balancing therapeutic benefits, societal perceptions, and accessibility. Understanding how environmental and organizational factors influence patient populations, treatment processes, and societal attitudes is vital for designing low-threshold, effective mental health services. The relocation of the KIS highlights these challenges, reflecting broader societal issues of inclusion and stigma. This study aims to explore how the ward’s location affects patient demographics, clinical outcomes, and perceptions among clinicians and the community.

### Aims and hypotheses

Following the relocation of the psychiatric KIS from the general somatic hospital USB to the psychiatric hospital UPK, this study examines pre- versus post-relocation differences among patients and clinicians.

We hypothesize that significant changes occurred in patient characteristics, including patient’s age and gender, the frequency of admissions of patients with specific diagnoses—particularly affective disorders (F3), anxiety, dissociative, stress-related, somatoform and other nonpsychotic mental disorders (F4) and personality disorders (F6), which constitute the most common diagnoses at the KIS—as well as in rates of involuntary admissions and transfers to other wards between the two settings. Based on prior research, we predict that patients at the psychiatric hospital will tend to be younger, more likely to be male, with increased admissions for personality disorders (F6), as well as higher rates of involuntary admissions and transfers.

Second, we will test whether patient- and clinician-reported treatment scores at admission were higher at the psychiatric hospital and whether treatment outcomes differed between the two patient cohorts.

Additionally, we will assess whether patients treated at the new location reported lower satisfaction.

Furthermore, we will test whether outpatient clinicians in surveys conducted pre- versus post-relocation rated the KIS locations and their treatment quality differently.

## Methods

### Setting and procedure

**Fig. 1:**
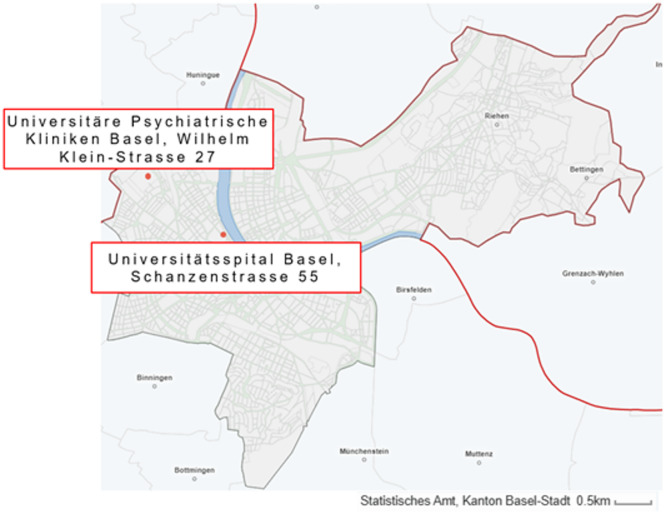
Current and former location of the KIS in Basel, Switzerland.

This observational study was initiated following the relocation of the KIS in Basel-City, Switzerland, on May 26, 2023. Figure 1 provides an overview of the current location of the KIS at the UPK on the outskirts of Basel and its former location at the USB in the city center, which are approximately 1.8 km apart.

The study involves two patient cohorts: the first includes patients treated at the former KIS location at USB from June 1, 2022, to May 25, 2023, and the second includes patients at the new KIS location within UPK from May 26, 2023, to May 31, 2024. The interdisciplinary staff of the KIS continued their work throughout the relocation period and at the new location.

To assess the perspective of referring clinicians, we invited outpatient psychiatrists and psychotherapists in Basel-City to participate in an online survey. The survey was conducted during two distinct three-month periods: the first from March to May 2023, prior to the relocation, and the second from March to May 2024, one year following the relocation.

All procedures performed in this study involving human participants were in accordance with the ethical standards of the institutional and national research committee and with the 1964 Helsinki Declaration and its later amendments. For the routine clinical data, which were anonymized and part of quality management of the UPK, a formal consent process was not required. Outpatient clinicians who participated anonymously in the online survey provided informed consent for their data to be used in publication. The study protocols were approved by the Ethics Committee of Northwestern Switzerland, and it was confirmed that no ethics committee vote was necessary for the analysis and publication of these data (EKNZ; Req-2024-00563_NZ_Stat_240424).

### Measurements

The data collection for this study comprised two datasets collected between June 2022 and May 2024: The first dataset consisted of routine data, including sociodemographic patient characteristics, patient-reported outcome measures (PROMs) and patient-reported experience measures (PREMs). Routine data collected during day-to-day operations were extracted and anonymized as part of quality management procedures and were made available for the study. The second dataset consists of a survey conducted among referring clinicians during two time periods.

Firstly, we counted the number of admissions to the KIS at both locations during the two separate one-year observation periods. Our analysis focused on several key outcome measures, including patient’s age and gender, involuntary admissions, and transfer rates to other wards of the UPK. We also examined the distribution of mental illness diagnoses among patients admitted to KIS using ICD-10 classifications (chapters F0 to F9)^56^.

Validated clinical measures were used, such as the Health of the Nation Outcome Scales (HoNOS)^57^, which evaluates various observable behaviors, including agitation, aggression, cognitive difficulties, and issues related to living conditions over the past seven days. HoNOS assessments were administered at both admission and discharge to evaluate clinical outcomes throughout the treatment process and integrate information from referring physicians, social workers, psychologists, and relatives, in addition to patient’s self-reported data. The German version of HoNOS (Andreas et al., 2007) consists of 12 items that assess mental health along with social and behavioral functioning. A 5-point Likert scale was employed, with 0 indicating “no impairment” and 4 signifying “severe impairment”, resulting in cumulative scores ranging from 0 to 48.

To include service users’ perspectives, we incorporated the Brief Symptom Checklist (BSCL)^58^ and the Münsterlingen Patient Satisfaction Questionnaire (MüPF), developed by the MüPF Benchmark Group and the Institute for Evaluation Research in Basel in 2008/2009.

The Brief Symptom Checklist (BSCL)^58^ is a self-report measurement filled out by patients (PROM) that consists of 53 items, which can be categorized into 9 subscales including “Depression”, “Phobic Anxiety”, and “Paranoia”. Additional symptoms, such as “poor appetite”, “difficulty falling asleep”, “thoughts of death or dying”, and “feelings of guilt”, which can manifest in various psychological disorders, are captured in the subscale labeled “Additional items”. Patients evaluate the presence of symptoms on a 5-point Likert scale, ranging from 0 (“not at all”) to 4 (“very strongly present”) within the past 7 days.

The patient satisfaction questionnaire MüPF (PREM) comprises 26-item assessing different treatment aspects, such as admission processes and patient involvement, with ratings on a 7-point Likert scale from 1 (“does not apply at all”) to 7 (“fully applies”). A “not answerable” option was provided for each item. The overall MüPF mean score was obtained by summing the item values and dividing the total score by the number of items.

Surveys among outpatient clinicians in Basel were conducted twice, each covering a three-month period: from March to May 2023 (pre-relocation) and from March to May 2024 (post-relocation). Invitations were distributed online through email distribution lists of professional associations, primarily targeting outpatient psychiatrists, psychotherapists, and general practitioners who could participate anonymously. A total of 50 clinicians participated in 2023, and 148 participated in 2024. The two surveys assessed clinicians’ perceptions of both the former and current locations of the KIS. Clinicians were asked to rate the quality of each location on a 10-point Likert scale, ranging from 1 (“not at all satisfied”) to 10 (“very satisfied”). Clinicians who had referred patients to the KIS during the three-month period were asked to evaluate their satisfaction with the treatment those patients received, also on a 10-point Likert scale. To get further insight into the referral process, the survey included open-ended text fields that enabled clinicians to specify reasons for not referring patients who required crisis intervention to the KIS. Finally, clinicians were encouraged to share their thoughts on the strengths and weaknesses of both KIS locations through open-ended responses.

### Data analysis

The clinical routine data were provided to the authors by the Institute for Evaluation Research Basel, a separate entity from UPK Basel. Patient data were anonymized through the assignment of a numerical ID. Statistical analysis was conducted using IBM SPSS Statistics 30.0^59^.

Descriptive statistics were used to summarize the sociodemographic and clinical characteristics of the study population, including means and standard deviations. To identify potential differences between the two patient cohorts, various mean comparisons were performed, including t-tests for independent samples, Mann-Whitney U tests, chi-squared tests, and Fisher’s Exact Test. The patient characteristics of particular interest in the comparison include age, gender distribution, the frequency of affective disorders (F3), anxiety, dissociative, stress-related, somatoform and other nonpsychotic mental disorders (F4), and personality disorders (F6), the number of involuntary admissions, and transfers to another ward. Furthermore, we compared clinician- and patient-reported treatment outcomes (HoNOS, BSCL) at admission and discharge, and patient satisfaction (MüPF). To account for multiple comparisons, the significance threshold was adjusted using a correction, dividing the conventional alpha level (*p* < 0.05) by the total number of tests conducted (*n* = 18). *P*-values remaining significant after this correction are indicated with an asterisk (*) in the tables depicting the results.

Information about other variables that were not further analyzed in our study can be found in Table 5 in the supplementary material.

In the referring clinician survey, the two groups of outpatient clinician were compared regarding their satisfaction with the treatment received by their referred patients at KIS using a Mann-Whitney U test. Within each clinician group, the Wilcoxon test was utilized to assess whether there were significant differences in evaluations of the KIS locations at USB and the UPK location. Finally, differences in location ratings identified in these analyses were compared between both groups using the Mann-Whitney U test. The Bonferroni correction was applied for those main outcomes of the clinician survey.

The open guiding questions at the end of the questionnaire could be answered in free text. These questions pertained to barriers regarding why patients were not referred to the KIS, experiences with the KIS, and sharing their thoughts on the strengths and weaknesses of both locations through open-ended responses. Based on the thematic synthesis^60^, the statements were analyzed in terms of content and summarized by describing recurring, relevant statements in thematic relation to the overarching terms. Finally, the statements were coded and the number of clinicians who had mentioned a coded statement was counted^61^.

## Results

### Patient characteristics

The main outcomes of our study are displayed in Table 1. Over the entire two-year study period, a total of 1311 cases involving 987 individual patients were managed at KIS. Between June 1, 2022, and May 26, 2023, a total of 625 cases were recorded. Post-relocation, from May 27, 2023, until May 31, 2024, 686 cases received treatment at the KIS.

**Table 1:** Overview and comparison of main patient demographic and treatment data at former and new location of the Kriseninterventionsstation (KIS).

Measure		Former location at Universitätsspital Basel (USB) (1st June 2022–25th May 2023)	New location at Universitäre Psychiatrische Kliniken Basel (UPK) (26th May 2023–31st May 2024)	t-test, chi-square test, Mann-Whitney U test, or Fisher Exact Test (*p* value)	Effect size (Cohen’s d, Pearson’s *r* or Phi Φ)
Number of cases		*n* = 625	*n* = 686		
*Sociodemographic characteristics*					
Age (years)		40.4 ± 14.5	38.0 ± 14.0	*T* = 2.994, *p* = 0.003	*d* **≈** 0.166
Female gender		413 (66.1%)	409 (59.6%)	*Χ*^2^ = 5.834, *p* = 0.016	φ ≈ -0.067
*Main diagnoses*					
F3: Mood [affective] disorders		195 (31.2%)	140 (20.4%)	*Χ*^2^ = 20.022, *p* < 0.001 *	φ ≈ − 0.124
F4: Anxiety, dissociative, stress-related, somatoform and other nonpsychotic mental disorders		224 (35.8%)	289 (42.1%)	*Χ*^2^ = 5.429, *p* = 0.02	φ ≈ 0.064
F6: Disorders of adult personality and behavior		133 (21.3%)	150 (21.9%)	*Χ*^2^ = 0.066, *p* = 0.797	φ ≈ 0.007
*Type of entry*					
Involuntary admissions		0 (0.0%)	4 (0.6%)	*Χ*^2^ = 3.693, *p* = 0.073	φ ≈ 0.053
*Transfers*					
Transfer to another inpatient station		65 (10.4%)	105 (15.3%)	*Χ*^2^ = 6.975, *p* = 0.008	φ ≈ 0.073
*Health of the Nation Outcome Scales (HoNOS) (0–48)*					
HoNOS at admission		13.6 ± 5.2 (*n* = 573)	15.3 ± 5.0 (*n* = 582)	*U* = 200,421, Z = 5.950, *p* = 0.946	*r* ≈ 0.175
HoNOS at discharge		9.1 ± 5.8 (*n* = 540)	9.8 ± 5.3 (*n* = 543)	*U* = 157,454, Z = 2.110, *p* = 0.595	*r* **≈** 0.064
HoNOS mean difference		4.3 ± 5.0	5.4 *±* 4.9	*U* = 171,768, Z = 4.896, *p* = 0.482	*r* ≈ 0.222
*Brief Symptom Checklist (BSCL) (0–4)*					
Global BSCL at admission		1.8 ± 0.7 (*n* = 275)	1.7 ± 0.7 (*n* = 284)	*U* = 37,788, Z = -0.661, *p* = 0.247	*r* ≈ 0.028
Global BSCL at discharge		1.4 ± 0.7 (*n* = 134)	1.3 ± 0.7 (*n* = 140)	*U* = 8055.5, Z = − 2.02, *p* = 0.043	*r* ≈ 0.122
*Patient satisfaction (MüPF) (1–7)*					
MüPF total score		5.2 ± 1.1 (*n* = 103)	4.6 ± 1.3 (*n* = 99)	*U* = 3728, Z = − 3.300, *p* < 0.001 *	*r ≈* 0.23

Remarks: Total number of cases are shown. In the HoNOS and BSCL rating scales, “0” indicates the best possible rating. In the MüPF rating scale, “1” indicates the worst possible rating. The significance level of *p* < 0.05 was divided by the number of tests conducted (18). The *p*-values that remain significant after correction are marked with an asterisk (*). Effect size interpretation: small effect (*d* = 0.2; Φ = 0.1; *r* < 0.3), medium effect (*d =* 0.5; Φ = 0.3; *r <* 0.5), large effect (*d =* 0.8; Φ = 0.5; *r > ≈* 0.5).

The proportion of male patients was 33.9% at the USB and 40.4% at the UPK, and did not differ. The mean age of the admitted patients was 40.4 ± 14.5 years pre-relocation and did not differ from the mean age of 38.0 ± 14.0 years post-relocation.

The three most common types of main diagnoses of patients admitted to KIS under the ICD-10 classification were affective disorders (F3), anxiety, dissociative, stress-related, somatoform and other nonpsychotic mental disorders (F4), and personality disorders (F6). Prior to relocation, 194 (31.2%) cases were classified as F3. At the new KIS location at the psychiatric hospital, the proportion of cases with an affective disorder (F3) diagnosis was lower - at 20.2% (140 cases), representing a small effect size (Φ = − 0.124). The frequencies of stress-related (F4) and personality disorders (F6) were comparable across locations, with F4 being the most common at both locations, with 224 (35.8%) at USB and 289 cases (42.1%) at UPK. 133 (21.3%) and 150 (21.9%) cases were diagnosed as F6.

No involuntary admissions to KIS were documented at USB, whereas four patients were involuntarily admitted at UPK. These numbers did not differ significantly. At the general hospital location, 65 cases (10.4%) were transferred from KIS to another inpatient unit of the UPK in comparison to 105 (15.3%) at the UPK. No significant difference was observed.

### Clinical outcomes

The analysis of the clinical outcomes of the two groups (before and after relocation) utilized the Health of the Nation Outcome Scales (HoNOS), with scores ranging from 0 (no symptoms present) to 48 (maximal symptom severity). Before the relocation, the mean admission score was 13.6 ± 5.2 (*n* = 573), compared to 15.3 ± 5.0 (*n* = 582) post-relocation. The achieved reduction in HoNOS scores was comparable in both cohorts. Thus, values at discharge were similar, with scores averaging 9.1 ± 5.8 (*n* = 540) in the USB cohort and 9.8 ± 5.3 in the UPK cohort. Treatment outcomes of both cohorts are presented in Table 1.

### Patient-reported outcome measures

The Brief Symptom Checklist (BSCL) scores of patients at admission did not reveal significant differences between the two patient cohorts. At the USB location, 275 patients reported a mean global BSCL score of 1.8 ± 0.7 at admission. Similarly, 284 patients treated at the UPK location reported an admission score of 1.7 ± 0.7. Both the global BSCL score and all nine subscales demonstrated similar reductions in symptom severity at both locations, resulting in discharge scores of 1.4 ± 0.7 at the USB (*n* = 134) and 1.3 ± 0.7 at the UPK (*n* = 140).

### Patient-reported experience measure

During the 12 months at the general hospital location, the average patient satisfaction score (MüPF) was 5.2 ± 1.1 (*n* = 103) on a scale ranging from 1 to 7. After the relocation to the psychiatric hospital, patient satisfaction was at 4.6 ± 1.3 (*n* = 99), a rating significantly lower than in the USB-treated cohort, with a small-to-medium effect size (*r* = 0.23). Patient satisfaction values for both KIS locations are shown in Table 1.

### Clinician survey

An overview of the quantitative analysis of the referring clinician survey is provided in Table 2. Information about the professions of the clinicians and the descriptive analysis of the importance of the KIS for their clinical work is given in Table 4 in the supplementary material.

**Table 2:** Quantitative results of the survey of referring clinicians from Basel-City on the perception of the former and new location of the KIS in 2023 and 2024.

Measure	Rating of location at general hospital (USB) (1–10)	Rating of location at psychiatric hospital (UPK) (1–10)	Mean difference for location rating (Wilcoxon test, *p*-value)	Effect size (Pearson’s *r*)
1st survey period pre-relocation (March 2023–May 2023)	9.5 ± 1.8 (*n* = 43)	4.3 ± 2.4 (*n* = 43)	5.2 (W = 28, *Z* = − 5.310, *p* < 0.001 *)	*r ≈* 0.81
2nd survey period post-relocation (March 2024–May 2024)	9.2 ± 1.8 (*n* = 141)	4.7 ± 2.3 (*n* = 142)	4.5 (W = 236, *Z* = − 9.228, *p* < 0.001 *)	*r ≈* 0.78

Remarks: Mean scores and standard deviations are shown. *n* = number of replies. In the rating scales, “1” indicates the worst possible rating. The significance level of *p* < 0.05 was divided by the number of tests conducted (18). The *p*-values that remain significant after correction are marked with an asterisk (*). Effect size interpretation: small effect (*r* < 0.3), medium effect (*r* < 0.5), large effect (*r* > ≈ 0.5).

**Table 3:** Qualitative results of the survey of referring clinicians from Basel-City on the perception of the former and new location of the KIS in 2023 and 2024.

Qualitative measure	1st survey period prior to the relocation of the KIS (March 2023–May 2023)	2nd survey period after the relocation of the KIS (March 2024–May 2024)
Number of referring clinicians	*n* = 50	*n* = 148
*General hospital (Universitätsspital Basel; USB)*		
Advantages of the location		
Central location and accessibility	*n* = 17 (34.0%)	*n* = 27 (18.2%)
Low treatment-seeking threshold	*n* = 12 (24.0%)	*n* = 14 (9.5%)
Reduced stigma	*n* = 11 (22.0%)	*n* = 21 (14.2%)
Connection to somatic hospital for assessments	*n* = 5 (10.0%)	*n* = 9 (6.1%)
Proximity to psychiatric outpatient clinic at Kornhausgasse	*n* = 1 (2.0%)	*n* = 1 (0.7%)
Inadequate environment	*n* = 0	*n* = 1 (0.7%)
Reduced social isolation by integrating patients into daily life	*n* = 0	*n* = 6 (4.1%)
Short waiting times	*n* = 0	*n* = 1 (0.7%)
Disadvantages of the location		
Challenges in transitioning to psychiatric clinic for referrals	*n* = 2 (4.0%)	*n* = 1 (0.7%)
Lack of collaboration with psychological and psychiatric staff	*n* = 1 (2.0%)	*n* = 0
Services not well known among target population	*n* = 1 (2.0%)	*n* = 0
Inadequate patient care	*n* = 0	*n* = 2 (1.4%)
Lack of synergy with psychiatric clinic	*n* = 0	*n* = 1 (0.7%)
Uncomfortable physical environment	*n* = 0	*n* = 1 (0.7%)
*Psychiatric hospital (Universitäre Psychiatrische Kliniken Basel; UPK)*		
Advantages of the location		
Improved integration into psychiatric services	*n* = 11 (22.0%)	*n* = 28 (18.9%)
Easier triage and transitions to psychiatric wards	*n* = 4 (8.0%)	*n* = 28 (18.9%)
Opportunity for destigmatization	*n* = 0	*n* = 9 (6.1%)
Protected, quiet, and aesthetically pleasing park area	*n* = 0	*n* = 10 (6.8%)
Disadvantages of the location		
Difficult accessibility in outer city area	*n* = 8 (16.0%)	*n* = 30 (20.3%)
Increased stigma	*n* = 7 (14.0%)	*n* = 22 (14.9%)
Increased barriers to seeking help	*n* = 2 (4.0%)	*n* = 17 (11.5%)
Feelings of social exclusion	*n* = 0	*n* = 2 (1.4%)
Sterile atmosphere within KIS building	*n* = 0	*n* = 1 (0.7%)
Separation of psychiatric and somatic care	*n* = 1 (2.0%)	*n* = 1 (0.7%)
Complications in the triage process	*n* = 0	*n* = 1 (0.7%)

Remarks: Mean scores and standard deviations are shown. *n* = number of replies. Percentage refers to the absolute number of clinicians participating in the survey period. Multiple replies per participant were possible.

The referring clinicians in both survey groups rated both KIS locations on a scale from 1 to 10. Evaluations of the former location at the general hospital were 9.5 ± 1.8 (*n* = 43) in 2023, and 9.2 ± 1.8 (*n* = 141) in 2024, post-relocation and thus retrospectively. The new location at the psychiatric hospital was rated at 4.3 ± 2.4 (*n* = 41) in 2023 and at 4.7 ± 2.3 (*n* = 142) in 2024. In both clinician groups, the rating difference in favor of the location at the general hospital was rated significantly better, corresponding to big effect sizes (*r* = 0.81 in 2023 and *r* = 0.78 in 2023). Ratings for each location between the two survey periods did not differ significantly.

Clinicians who referred at least one patient to the KIS rated their satisfaction with the treatment their patients received at the KIS. Satisfaction with patient treatment was similar in both groups, with a mean of 7.6 ± 2.5 (*n* = 10) in 2023, and 7.0 ± 2.5 (*n* = 67) in 2024.

Advantages or disadvantages for both the former location at the general hospital and the new location at the psychiatric hospital were elaborated by the clinicians in both survey periods through open text fields, with multiple mentions allowed (s. Table 3). Clinicians noted that the general hospital’s central location and accessibility (18.2–34.0%), lower treatment threshold (9.5–22.0%), reduced stigma (14.2–22.0%), and connections to somatic hospital assessments (6.1–10.0%) were key advantages. Few disadvantages were mentioned for USB, mainly limited collaboration with psychiatric staff (2.0%) and challenges during inpatient transitions (0.7–4.0%). For the psychiatric hospital location, benefits included a calming, aesthetically pleasing park area (6.8% in 2024), better integration into psychiatric services (18.9–22.0%), easier triage and transitions (8.0–18.9%), and potential for destigmatization (6.1% in 2024). However, clinicians highlighted accessibility issues due to its outer city location (16.0–20.3%), and about 14% expressed concerns about increased stigma and higher treatment-seeking thresholds (4.0–11.5%).

Referring clinicians from both survey groups provided insights into why they did not refer patients to the KIS. In 2023, the most frequently cited reasons included a lack of therapeutic indication (*n* = 8, 16.0%), patient did not want to be hospitalized (*n* = 11, 22.0%), patient concerns regarding KIS treatment (*n* = 3, 6.0%), and organizational obstacles for out-of-canton referrals (*n* = 1, 2.0%). In 2024, clinicians reported several reasons for not making referrals, including patients’ preferences for outpatient treatments or alternative solutions for crises (*n* = 21, 14.2%), patient concerns about the stigma associated with psychiatric hospitals (*n* = 15, 10.1%), and a lack of indication for an inpatient crisis intervention (*n* = 14, 9.5%). Three clinicians criticized the admission process as a barrier for referrals. Additionally, five clinicians indicated that their patients were referred to KIS through other institutions or sought out KIS independently without a referral.

## Discussion

Following the relocation of a psychiatric crisis intervention ward from a centrally located general to a psychiatric hospital on the outskirts of Basel, we did not observe significant differences across most examined variables between the two patient cohorts. However, the primary observed changes were a lower patient-reported satisfaction among the cohort at the psychiatric hospital and a critical perception of the ward’s location by outpatient clinicians, who may, in turn, influence patient referrals. The key demographic and diagnostic characteristics of the two observed patient cohorts did not exhibit differences except for the lower frequency of affective disorders (F3) among individuals seeking help at the KIS after its relocation to the University Psychiatric Clinics (UPK), which, however, corresponds to a small effect. Involuntary admissions rarely occurred during the observation period, and the number of transfers was comparable. Overall, it cannot be concluded from the data that the new location at the psychiatric hospital may be attracting a different segment of the population. An extension of the observation period is recommended in order to establish any demographic trends in the patient populations.

Notably, patients at both sites of the KIS exhibited lower clinician-reported HoNOS scores (mean scores of 13.6 and 15.3) compared to the average HoNOS score of 18.5 observed across all UPK wards in 2024. Additionally, these scores were below the average HoNOS of 19.9 at admission reported across all Swiss clinics in 2024, with a discharge score of 11.5^62^. The discharge HoNOS scores at the KIS were comparable to those across UPK, averaging 10.3.

Regarding the BSCL, the mean scores in our sample were higher both at admission (1.8, sum: 95.4; 1.7, sum: 90.1) and at discharge (1.4, sum: 74.2; 1.3, sum: 68.9) compared to the reference data from all Swiss psychiatric clinics in 2024, which reported 1.4 (sum: 74.3) at admission and 0.8 (sum: 34.1) at discharge^62^. This could be interpreted as consistent with the concept of timely, limited crisis intervention aimed at rapid symptom reduction, while more severely ill patients might be transferred to other specialized psychiatric wards. In total, discharge scores exhibited a similar degree of reduction across both locations, indicating that treatment effectiveness was similar regardless of the setting. This observation supports the hypothesis that the fundamental therapeutic strategies employed in the KIS may be more influential than contextual variations.

However, the lower patient-reported satisfaction ratings following the relocation raise concerns. While the patient satisfaction scores were notably lower among the cohort treated post-relocation, it is important to recognize that patient satisfaction can be influenced by a variety of factors. It could suggest that the relocation to the psychiatric hospital may have temporarily affected the patient experience, but this does not necessarily indicate a fundamental decline in care quality, especially given the stable treatment outcomes observed in both clinician assessments (HoNOS) and patient self-reports (BSCL). The lower satisfaction may also be a transient effect related to the relocation and the subsequent adjustment period for the treatment team, the loss of positive locational factors at the USB, such as its central position in Basel-City and a neutral setting, and potential prejudices against the psychiatric hospital. Additionally, patients may have been influenced by media reports regarding the opposition to the relocation of the KIS^26^, leading to negative priming effects in their evaluations^63–65^. The reduced patient satisfaction may also be associated with the negative perceptions of outpatient providers identified in the referring clinician survey, as patients may have sensed reservations from their providers regarding the new location within the psychiatric hospital. Maintaining a patient-centered approach during such relocations is paramount, as dissatisfaction can influence future help-seeking behaviors and overall treatment outcomes^66,67^. Therefore, ongoing monitoring of patient outcomes is necessary. However, the total number of cases admitted to the KIS during the two-year observation period reached 1,311, underscoring its significance as a crisis intervention resource in Basel-City.

Quantitative and qualitative evaluations by referring clinicians indicate that both clinician groups from the 2023 and 2024 surveys preferred the USB location to the UPK location. However, clinician’s ratings of the new KIS location in the psychiatric hospital were significantly worse than the perception of the former general hospital location within both survey groups. Numerous outpatient therapists and treatment providers in Basel (s. Table 3), view the relocation of the KIS to UPK as disadvantageous, citing fears that patients may develop negative perceptions of inpatient treatment, apprehensions about interacting with other individuals with mental illnesses, and concerns about potential stigmatization. This highlights a substantial need for increased education and information regarding the KIS and the psychiatric hospital in general and an ongoing dialogue between the KIS and referring professionals, to improve collaborative practices as well as to ensure that the KIS is recognized as a valuable option during crises. Furthermore, several clinicians (22.0% in 2023 and 14.2% in 2024), expressed concerns regarding their patients’ preferences for alternative solutions outside of inpatient psychiatric settings during crises.

Nonetheless, some advantages of the new location at the psychiatric hospital were also mentioned by the clinicians. These included better integration and easier triage within the psychiatric services, positive contextual factors, and opportunities for reducing the stigma surrounding the psychiatric hospital. Interestingly, among those clinicians who had referred patients to the KIS, the treatment satisfaction ratings showed no significant differences and remained high. This negative perception among referring clinicians likely reflects their underlying beliefs about the efficacy of care and the holistic environment of the treatment setting, which may or may not be justified given the absence of significant differences in treatment outcomes. Such perceptions can have profound implications on referral patterns and ultimately influence patient access to necessary psychiatric services.

The findings of this study raise important questions regarding the implications of spatial considerations in mental health service delivery^22,23,25,27^. While specialized psychiatric facilities can offer enhanced clinical expertise and resources, they may inadvertently create barriers for certain individuals due to perceived stigma and accessibility issues. The current findings highlight the necessity of balancing accessibility, therapeutic environment, and societal perception - factors that are deeply intertwined with historical debates on the “right” placement for psychiatric services^28,29^.

Future research should investigate the long-term consequences of such relocations on patient outcomes and satisfaction, particularly focusing on the role of stigma and accessibility in influencing treatment pathways. Additionally, assessing whether media reports that highlight the benefits of the relocation - such as sustained symptom reduction in patients, better integration into psychiatric services and the calming park area - alter the attitudes of the public, patients and clinicians toward the new location, could provide important insights. Qualitative studies exploring patient experiences may offer a deeper understanding of the complexities involved in care transitions within psychiatric settings. An aspect not addressed in our current study, but worthy of future exploration, is the perceived differences in working conditions and team dynamics from the perspective of the treatment staff at the two locations.

### Strengths and limitations

A notable strength of this study lies in its pioneering exploration of the effects of relocating a psychiatric unit - not merely in terms of physical space, but within a different healthcare setting. A primary methodological strength is its multi-method observational design, which combines quantitative with qualitative data pre- and post-relocation to provide a comprehensive understanding of the KIS relocation’s potential effects, including whether the change in ward setting was linked to patient characteristics, admission patterns, clinical outcomes, and stakeholder perceptions. The extended two-year data collection period enhances the robustness of the findings by mitigating the influence of short-term anomalies and potential confounding factors related to the relocation process.

However, limitations are also present, particularly the potential biases inherent in self-reported data from both patients and clinicians. The reliance on self-reported data from both patients and clinicians introduces potential biases, including subjective perceptions and social desirability effects^68,69^. In particular, the two clinician surveys, conducted anonymously, bear the risk of repeated participation by the same individuals across different periods, potentially skewing results. The large sample size can be seen as a strength of the study; however, it also increases the likelihood that small effects which may become statistically significant^70^. The study was conducted at a single center, focusing exclusively on the Crisis Intervention Ward in Basel-City. Therefore, replication for external validation of the study’s findings in other settings is necessary. A multicenter approach would have been beneficial, as it could have provided a broader perspective on the system changes in healthcare delivery. However, implementing a multicenter study would be complex and challenging due to the extensive changes required in clinical practice within the healthcare system. Consequently, while the findings offer valuable insights into the immediate changes following the KIS relocation, further longitudinal research is essential to fully understand the lasting implications of this change of setting in mental health service delivery.

## Conclusion

This comprehensive analysis of the KIS before and after its relocation to the psychiatric hospital in May 2023 demonstrates that clinical outcomes, as measured by HoNOS and BSCL, remained stable across both settings, indicating consistent treatment efficacy. Patient characteristics, including demographics, referral patterns, and diagnostic profiles, showed only minor variations. Importantly, patient satisfaction was significantly lower at the new KIS location in the psychiatric hospital, and clinicians expressed a preference for the previous general hospital setting, citing reduced accessibility and stigma associated with the psychiatric hospital location as potential disadvantages. Lower patient satisfaction and the increased treatment-seeking thresholds perceived by outpatient clinicians are likely related to the relocation or attitudes toward the psychiatric hospital as the new KIS location. Several plausible explanations include loss of positive factors associated with the former location at the general hospital, an adjustment period, negative media coverage surrounding the relocation, and potential biases or prejudices. However, clinical outcome measures did not indicate deterioration in treatment quality. Notably, the relocation of the KIS also offers opportunities, including potential destigmatization and the creation of a more secure therapeutic environment. As the placement of psychiatric services continues to reflect broader societal values, debates around accessibility, stigma reduction strategies, and community acceptance remain influential in policy decisions.

In summary, the relocation of the KIS to the psychiatric hospital embodies both challenges and opportunities within psychiatric care. While the intent of the relocation is to improve the quality of crisis interventions, it is crucial to consider the socio-environmental factors influencing patient access and perceptions. As psychiatric services continue to evolve, prioritizing patient-centered practices and addressing the complex nature of stigma will be vital in ensuring that crisis interventions remain effective and accessible to all individuals in need. Future research should focus on the long-term implications of this relocation on patient outcomes and satisfaction, thereby informing best practices in mental health care delivery.

## Supplementary Information

Below is the link to the electronic supplementary material.

Supplementary Material 1

## Data Availability

The data supporting the results reported in this article are available upon request from the authors.
