# Supplementary material for: The relocation of a psychiatric crisis intervention unit from a somatic to a psychiatric hospital: a multi-method observational study
Source: Sci Rep. 2026 Jun 19;16:20742. doi: 10.1038/s41598-026-57920-5 (PMC13338241; doi:10.1038/s41598-026-57920-5)
Supplement: Supplementary file 1 — Supplementary Material 1 [file 41598_2026_57920_MOESM1_ESM.docx]

Table 4. Overview of explorative analysis of patient demographic and treatment data at former and new location of the Kriseninterventionsstation (KIS).

| Measure | | | Former location at  Universitätsspital Basel (USB)  (1st June 2022 – 25th May 2023) | | | New location at  Universitäre Psychiatrische Kliniken Basel (UPK) (26^th^ May 2023 – 31st May 2024) | | |
| --- | --- | --- | --- | --- | --- | --- | --- | --- |
| **Number of cases** | | | *n* = 625 | | | *n* = 686 | | |
| Occupancy rate | | | 88.7% | | | 95.6% | | |
| Swiss nationality | | | 422 (67.5%) | | | 460 (67.1%) | | |
| **Main diagnoses** | | | *n* = 625 | | | *n* = 686 | | |
| **F1:** Mental and behavioral disorders due to psychoactive substance use | | | 22 (3.5%) | | | 30 (4.4%) | | |
| **F2:** Schizophrenia, schizotypal and delusional disorders | | | 27 (5.9%) | | | 51 (7.4%) | | |
| **F5:** Behavioral syndromes associated with physiological disturbances and physical factors | | | 0 (0.0%) | | | 2 (0.2%) | | |
| **F7:** Mental retardation | | | 1 (0.2%) | | | 0 (0.0%) | | |
| **F8:** Disorders of psychological development | | | 4 (0.6%) | | | 12 (1.7%) | | |
| **F9:** Behavioral and emotional disorders with onset usually occurring in childhood and adolescence | | | 2 (0.3%) | | | 1 (0.1%) | | |
| **Type of entry** | | | *n* = 625 | | | *n* = 686 | | |
| Patient’s own initiative | | | 220 (35.2%) | | | 289 (42.1%) | | |
| Relatives, volunteers, laypersons | | | 2 (0.3%) | | | 6 (0.9%) | | |
| Emergency medical services | | | 2 (0.3%) | | | 6 (0.9%) | | |
| Physician | | | 46 (7.4%) | | | 39 (5.7%) | | |
| Psychiatrist | | | 67 (10.7%) | | | 62 (9.0%) | | |
| General hospital, somatic hospital | | | 63 (10.1%) | | | 72 (10.5%) | | |
| Outpatient psychiatric institution of UPK | | | 173 (27.7%) | | | 163 (23.8%) | | |
| Inpatient psychiatric institution of UPK | | | 4 (0.6%) | | | 5 (0.7%) | | |
| Other outpatient psychiatric institution | | | 18 (2.9%) | | | 11 (1.6%) | | |
| Other inpatient psychiatric institution | | | 2 (0.3%) | | | 4 (0.6%) | | |
| Non-medical professionals (e.g psychotherapists) | | | 8 (1.3%) | | | 12 (1.7%) | | |
| Other | | | 5 (0.8%) | | | 6 (0.9%) | | |
| Unknown | | | 9 (1.4%) | | | 5 (0.7%) | | |
| **Transfers** | | | *n* = 625 | | | *n* = 686 | | |
| Transfer from another inpatient station | | | 9 (1.6%) | | | 11 (1.9%) | | |
| **Type of discharge** | | | *n* = 576 | | | *n* = 586 | | |
| Both sides agreed on discharge | | | 558 (96.9%) | | | 564 (96.2%) | | |
| Discharge without physician’s consent | | | 11 (1.9%) | | | 15 (2.6%) | | |
| Discharge physician’s initiative | | | 7 (1.2%) | | | 6 (1.0%) | | |
| Deceased | | | 0 (0.0%) | | | 1 (0.02%) | | |
| **Treatment duration (days)** | | | 5.2 ± 2.2 | | | 5.2 ± 2.1 | | |
| Global BSCL at admission | 1.8 ± 0.7 (*n* = 275) | | | 1.7 ± 0.7 (*n* = 284) | | |  |  |
| BSCL Somatization | 1.6 ± 0.9 | | | 1.5 ± 0.9 | | |  |  |
| BSCL Depression | 2.3 ± 1.0 | | | 2.3 ± 1.0 | | |  |  |
| BSCL Anxiety | 2.0 ± 0.9 | | | 1.9 ± 1.0 | | |  |  |
| BSCL Phobic fear | 1.5 ± 1.0 | | | 1.4 ± 1.0 | | |  |  |
| BSCL Aggressiveness | 1.5 ± 0.9 | | | 1.5 ± 0.9 | | |  |  |
| BSCL Paranoia | 1.4 ± 1.0 | | | 1.4 ± 1.1 | | |  |  |
| BSCL Psychoticism | 1.5 ± 0.9 | | | 1.5 ± 0.9 | | |  |  |
| BSCL Insecurity | 1.9 ± 1.0 | | | 2.0 ± 1.1 | | |  |  |
| BSCL Additional items | 2.1 ± 1.0 | | | 2.1 ± 0.9 | | |  |  |
| Global BSCL at discharge | 1.4 ± 0.7 (*n* = 134) | | | 1.3 ± 0.7 (*n* = 140) | | |  |  |
| BSCL Somatization | 1.1 ± 0.9 | | | 0.9 ± 0.8 | | |  |  |
| BSCL Depression | 1.8 ± 1.0 | | | 1.6 ± 0.9 | | |  |  |
| BSCL Anxiety | 1.6 ± 1.0 | | | 1.4 ± 0.9 | | |  |  |
| BSCL Phobic fear | 1.4 ± 1.1 | | | 1.1 ± 1.0 | | |  |  |
| BSCL Aggressiveness | 1.1 ± 0.8 | | | 1.0 ± 0.7 | | |  |  |
| BSCL Paranoia | 1.3 ± 1.0 | | | 1.0 ± 0.9 | | |  |  |
| BSCL Psychoticism | 1.3 ± 0.9 | | | 1.1 ± 0.8 | | |  |  |
| BSCL Insecurity | 1.6 ± 1.0 | | | 1.5 ± 0.9 | | |  |  |
| BSCL Additional items | 1.6 ± 0.9 | | | 1.6 ± 0.8 | | |  |  |
| MüPF total score | | 5.2 ± 1.1 (*n* = 103) | | | 4.6 ± 1.3 (*n* = 99) | | |  |
| Overall satisfaction and willingness to recommend | | 5.4 ± 1.6 | | | 4.5 ± 1.9 | | |  |
| Treatment | | 5.5 ± 0.9 | | | 5.1 ± 1.2 | | |  |
| Therapeutic alliance | | 5.4 ± 1.5 | | | 4.9 ± 1.5 | | |  |
| Participation | | 5.1 ± 1.4 | | | 4.4 ± 1.5 | | |  |
| Infrastructure | | 5.1 ± 1.3 | | | 4.8 ± 1.4 | | |  |
| Aftercare services | | 5.1 ± 1.9 | | | 4.2 ± 2.2 | | |  |

Remarks. Total number of cases, mean scores and standard deviations are shown.

Table 5. Descriptive results of the survey of referring clinicians from Basel-City on the perception of the former and new location of the KIS in 2023 and 2024.

| Measure | 1^st^ survey period prior to the relocation  (March 2023 – May 2023) | 2^nd^ survey period after the relocation  (March 2024 – May 2024) |
| --- | --- | --- |
| Number of referring clinicians | *n* = 50 | *n* = 148 |
| Profession |  |  |
| Outpatient psychotherapist for adults | *n* = 37 (74.0%)+ | *n* = 132 (89.2%)+ |
| Inpatient psychotherapist | *n* = 0 (0.0%) | *n* = 2 (1.4%) |
| Psychiatrist for adults | *n* = 13 (26.0%)+ | *n* = 30 (20.3%)+ |
| Psychiatrist for children and adolescents | *n* = 0 (0.0%) | *n =* 2 (1.4%)+ |
| Outpatient child and adolescent psychotherapist | *n* = 1 (2.0%) | *n* = 4 (2.7%)+ |
| Geriatric psychiatrist and psychotherapist | *n* = 0 (0.0%) | *n* = 1 (0.7%)+ |
| Psychological counseling and crisis intervention | *n* = 1 (2.0%) | *n* = 3 (2.0%)+ |
| Family, couples, and parenting counseling | *n* = 1 (2.0%) | *n* = 3 (2.0%)+ |
| Supervision and teaching | *n* = 0 (0.0%) | *n* = 1 (0.7%)+ |
| Missings | *n =* 5 (10.0%) | *n =* 2 (1.4%) |
| Importance of KIS for clinical work | | |
| Awareness of KIS | 7.7 ± 2.6 (*n* = 45) | 7.6 ± 2.4 (*n* = 145) |
| Rating of importance  (1-10) | 4.3 ± 2.8 (*n* = 44) | 4.7 ± 3.0 (*n* = 145) |
| Number of indications | 1.4 ± 3.2 (*n* = 42) | 1.6 ± 2.7 (*n* = 146) |
| Number of patients with concerns regarding the general hospital location (USB) | 0.4 ± 0.9 (*n* = 35) | 0.3 ± 0.6 (*n* = 129) |
| Number of patients with concerns regarding the psychiatric hospital location (UPK) | 0.7 ± 1.1 (*n* = 35) | 0.9 ± 2.2 (*n* = 125) |
| Number of referred patients to the KIS | 0.4 ± 1.6 (*n* = 42) | 0.4 ± 0.8 (*n* = 144) |

Remarks. Mean scores and standard deviations are shown. *n* = number of replies. + indicate that a portion of the practitioners who participated in the survey reported multiple professions. In the rating scales, “1” indicates the worst possible rating.
